# Pylorus-preserving pancreatoduodenectomy for pancreatic head cancer after surgery for esophageal cancer with gastric tube reconstruction in a long-term survivor: A case report

**DOI:** 10.1016/j.ijscr.2019.01.024

**Published:** 2019-01-30

**Authors:** Takashi Orii, Masaki Yoshimura, Hiroe Kitahara, Yukihiko Karasawa

**Affiliations:** Showa Inan General Hospital, Akaho 3230, Komagane, Nagano, 399-4117, Japan

**Keywords:** Pylorus-preserving pancreatoduodenectomy, Gastroduodenal artery, Right gastroepiploic artery, Gastric tube, Esophagectomy

## Abstract

**Introduction:**

To maintain the blood circulation of the gastric tube in pylorus-preserving pancreatoduodenectomy (PPPD) for periampullary cancer after esophagectomy for esophageal cancer, it is necessary to preserve the gastroduodenal artery and its branch, the right gastroepiploic artery, which are usually resected for more convenient, complete, and effective lymph node dissection. Here we report the case of a patient with a postoperative survival period of more than 5 years.

**Presentation of case:**

A 79-year-old man, who underwent subtotal esophagectomy and gastric tube reconstruction 11 years ago, was diagnosed with pancreatic head cancer during routine examination 5 years after the esophageal surgery. After placement of a coronary artery stent for an arterial branch stenosis incidentally found during preoperative screening electrocardiogram, he underwent pancreatic surgery. As the tumor did not extend to the anterior surface of the pancreas and as there were no swollen lymph nodes in the area, the gastroduodenal artery, the gastroepiploic artery and vein could be preserved. Elective PPPD was conducted without incident, and good preoperative gastric tube circulation was maintained postoperatively. Reconstruction was performed according to the modified Child procedure with duct-to-mucosa stentless pancreaticojejunostomy. The postoperative course was uneventful and though it took the patient a long time to overcome the physical decline, he remains alive with no recurrent disease over 5 years post-operation.

**Conclusion:**

Although PPPD may be performed after esophagectomy with gastric tube reconstruction, it is still unclear how the risk of recurrence is affected. Therefore, the indications of this procedure should always be carefully considered.

## Highlights

• Gastric tube after esophagectomy needs blood circulation of gastroepiploic artery.
• Gastroduodenal artery is always resected in pancreatoduodenectomy.
• Preservation of gastroduodenal artery may introduce recurrence of pancreatic cancer.
• Long survival after pancreatoduodenectomy is possible even after esophageal surgery.

## 1 Introduction

Recently, there has been an increase in the number of patients with double cancers occurring simultaneously or metachronously and in the treatment options. Pancreatoduodenectomy (PD) is the standard surgery for resectable periampullary cancer and involves resection of the gastroduodenal artery (GDA) and its branches such as the right gastroepiploic artery (RGEA) to allow for complete dissection of lymph vessels and nodes. However, when the cancer occurs in patients who have had esophageal cancer surgery, we have to decide whether maintaining the gastric tube circulation or aiming for a definitive surgical cure for the cancer.

Here we report the case of a patient who underwent PD without GDA resection for pancreatic head cancer after surgery for lower esophageal cancer with a postoperative survival time of more than 5 years.

The work has been reported in line with the SCARE criteria [1].

## 2 Case presentation

A 79-year-old man, who underwent subtotal esophagectomy and reconstruction using a gastric tube 11 years ago, visited a primary care doctor with abdominal pain and no previous signs of disease recurrence over a period of 5 years. Ultrasonography revealed dilatation of the biliary tree and he was referred to our hospital.

Laboratory test showed no hepatorenal or hematological abnormalities. It was observed that the titer of carbohydrate antigen 19-9 increased slightly to 54.1 U/ml, though carcinoembryonic antigen, DUPAN-2, and Span-1 were within normal range.

**Fig. 1:**
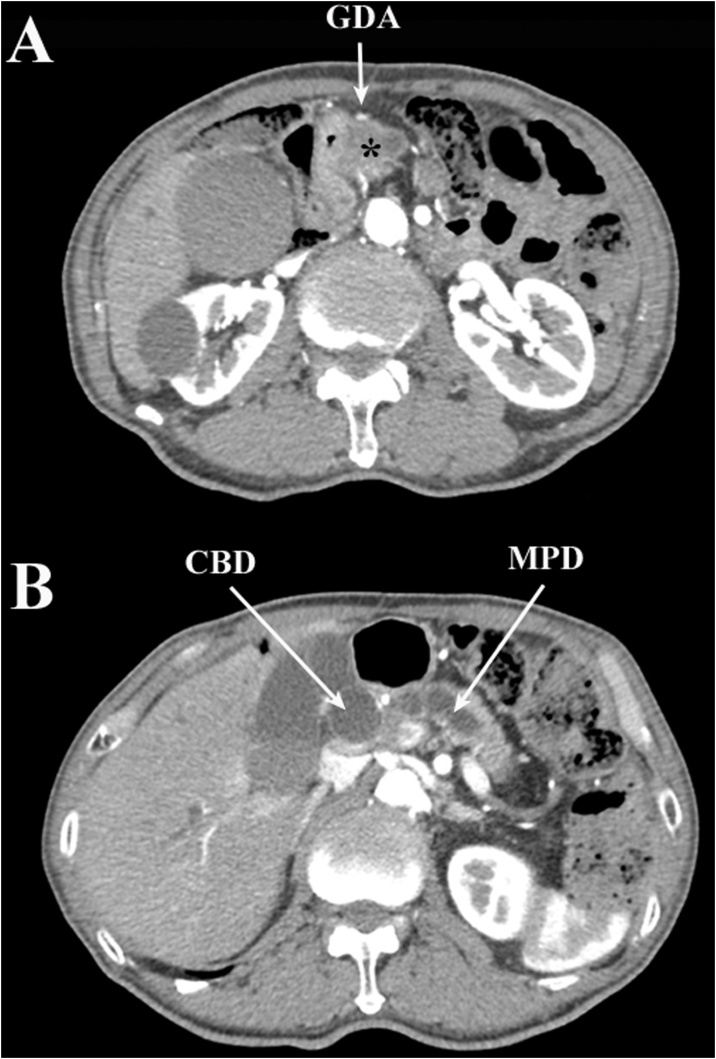
Scans of early-phase preoperative computed tomography with contrast medium; A: the gastroduodenal artery passing over the surface of the pancreatic head without tumor invasion; the asterisk indicates the pancreatic head cancer; B: the common bile duct and main pancreatic duct are considerably dilated.

**Fig. 2:**
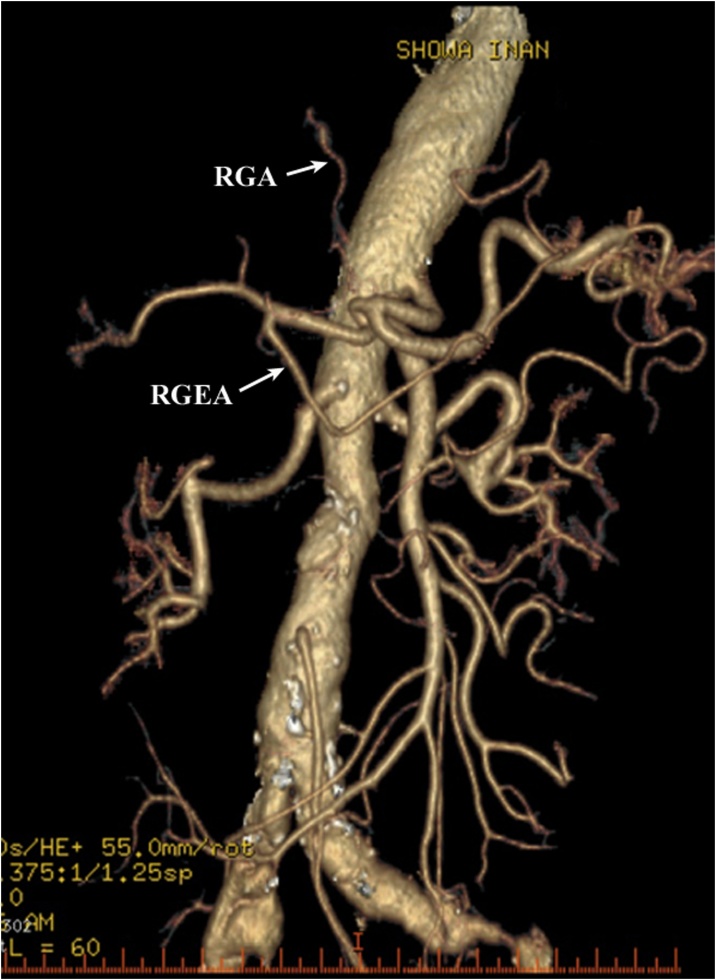
Preoperative 3-dimensional angiography reconstructed from the computed tomography scan; the right gastroepiploic and right gastric arteries are shown supplying blood to the gastric tube.

**Fig. 3:**
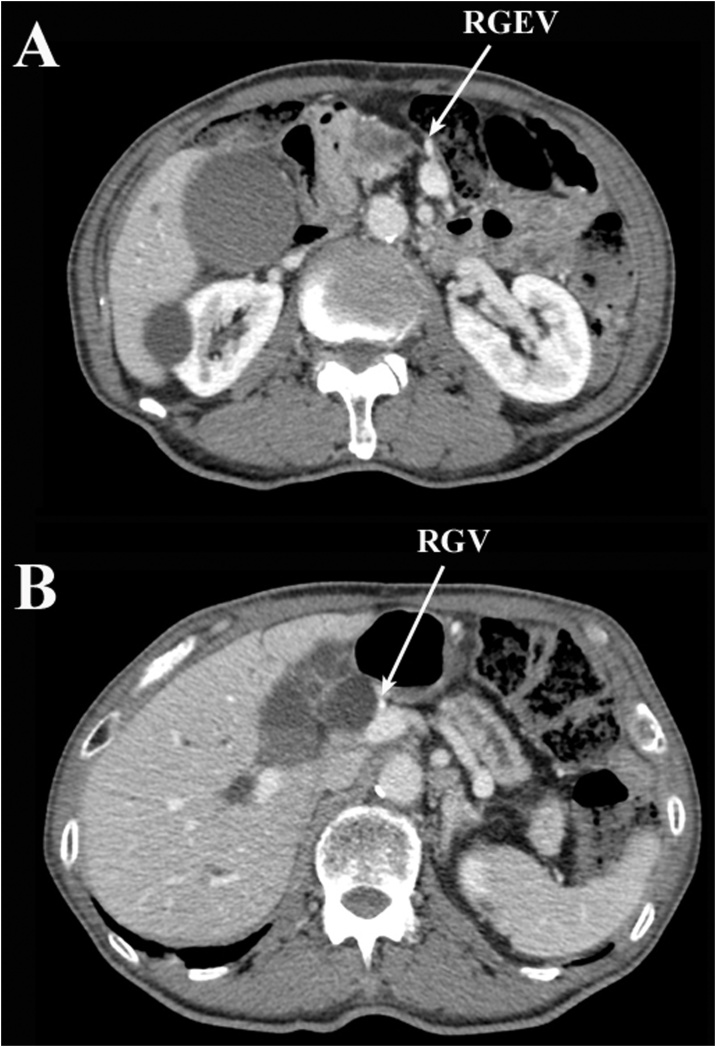
Scans of delayed-phase preoperative computed tomography with contrast medium; A and B: the right gastroepiploic and right gastric veins are patent and act as drainage veins for the gastric tube.

Contrast-enhanced computerized tomography (CT) showed a low-density area of 20 mm in the pancreatic head at the convergence of the dilated common bile duct and the main pancreatic duct. The tumor did not reach the surface of the pancreas and did not invade the GDA (Fig. 1). The patency of the RGEA, right gastroepiploic vein (RGEV), right gastric artery (RGA), and right gastric vein (RGV) were confirmed (Fig. 2, Fig. 3). There was no distant metastasis.

Magnetic resonance imaging showed a tumor with irregularly low-intensity on T2 weighted image, mild low-intensity on T1 weighted image, and gradual enhancement with contrast medium from the margin.

A combination of positron emission tomography with fluoro-2-deoxyglucose and CT confirmed the tumor as a thin uptake area with a maximum standard uptake value of 2.6.

Based on the above findings, the patient was diagnosed with resectable pancreatic head cancer. However, as electrocardiogram revealed a complete left bundle branch block and coronary angiogram showed a 99% stenosis of a coronary artery branch, a coronary artery stent was placed. Obstructive jaundice occurred a few days following the stenting and an endoscopic biliary stent was placed. The operation for the pancreatic cancer was performed a month after biliary drainage.

### 2.1 Surgical procedure

Following laparotomy by upper midline incision, peritoneal exploration was conducted but revealed no peritoneal metastases. Although palpable as a hard mass of about 3 cm in diameter, the tumor was not observable on the anterior surface of the pancreatic head, so the RGEA and RGEV could be preserved.

Dissection of the paraaortic lymph nodes (#16b1-inter, -pre, and -lat) was performed using Kocher’s maneuver, and the nodes were perioperatively confirmed by pathology to be negative for cancer cells. Henle’s trunk was exposed and the inferior pancreaticoduodenal vein and accessory right colic vein, which are tributaries of the trunk, were resected. However, the RGEV, another tributary of Henle’s trunk, was preserved.

The GDA and proper hepatic artery were confirmed after resection of the supraduodenal arteries following the dissection of the lymph nodes around the common hepatic artery (#8a and 8p). After the gallbladder was detached from its hepatic bed, the common hepatic duct was skeletonized and disconnected. The lymph nodes of the hepatoduodenal ligament (#12a, 12p, and 12b) were dissected. After adequately separating the GDA from the posterior surface of the duodenum, the duodenal bulb was cut 2 cm distal to the pyloric ring using GIA 60.

The jejunum was cut about 15 cm distal to the Treitz ligament and the mesentery was resected with the first jejunal artery (J1A) and vein. After skeletonizing the common root of the J1A and the inferior pancreaticoduodenal artery from the superior mesenteric artery (SMA), the root was resected. The proximal cut end of the jejunum was then drawn out to the right and passed posterior to the SMA and the superior mesenteric vein (SMV), and some small SMV branches from the pancreatic head were resected.

**Fig. 4:**
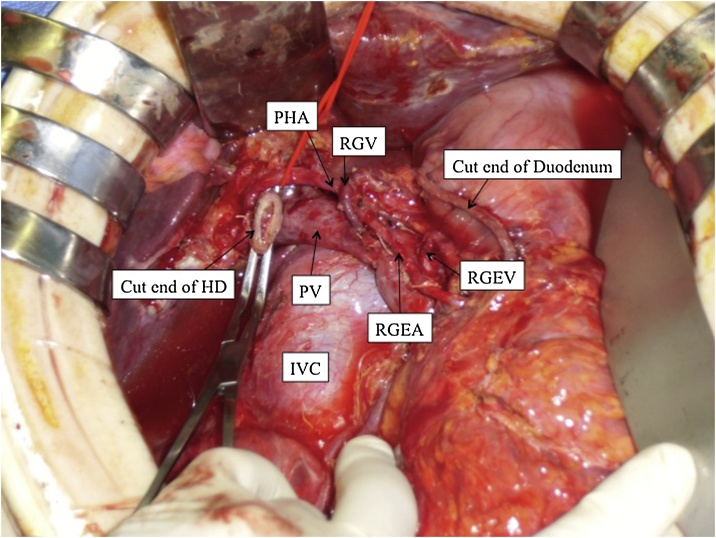
The operative view after pylorus-preserving pancreatoduodenectomy; it shows the preservation of vessels providing blood circulation to the gastric tube. HD; hepatic duct, PV; portal vein, IVC; inferior vena cava.

The GDA-RGEA was completely separated from the pancreatic head by resecting 3 small branches and the posterior superior pancreaticoduodenal artery. After confirming the inflow site of the RGV, the pancreatic parenchyma was cut with a scalpel along the left margin of the portal vein (PV)-SMV following the tunneling method. Pylorus-preserving pancreatoduodenectomy (PPPD) was then completed after dissecting the lymph nodes along the SMA (#14p, 14d), and the plexus of the pancreatic head and the right half of the SMA (Fig. 4).

Although the reconstruction was performed based on the modified Child procedure, Roux-en-Y procedure was adopted because, in aiming to preserve the RGA, RGV, RGEA and RGEV, the degree of freedom was limited. Stents were not used in duct-to-mucosa pancreaticojejunostomy and hepaticojejunostomy. Prophylactic closed drains with continuous aspiration were placed around hepaticojejunostomy and pancreaticojejunostomy.

The histopathological examination of the resected specimen showed that, although the moderately differentiated invasive ductal carcinoma reached the bile duct and duodenum and metastasized to 1 of the 18 resected regional lymph nodes, the tumor did not extend to the surface of the pancreas. The tumor was stage II according to the TNM classification of the Union for International Cancer Control (7th edition).

Although postoperative physical rehabilitation took a relatively long time because the patient had a poor preoperative physical condition (American Society of Anesthesiologists performance status 3), he did not experience complications like pancreatic fistula or surgical site infection. He was discharged on postoperative day 36 and remains alive with no disease recurrence 5 years and 3 months after the surgery.

## 3 Discussion

The prognosis of periampullary cancers such as pancreatic head cancer, bile duct cancer, and cancer of papilla Vater, is poor and complete resection is the only definitive treatment. Since radical resection involves complete dissection of lymph nodes, neural plexuses and surrounding connective tissue, it is important to also resect the nutritional artery and drainage vein.

In contrast, it is essential, in the surgery for esophageal cancer, to preserve the RGEA to maintain the blood circulation and status of the gastric tube as a reconstructed organ.

In recent years, along with the increase in lifespan, the number of the patients with double cancers occurring non-concomitantly has increased. While deliberation on whether to preserve or resect a vessel in a patient is ongoing, the chance to treat the cancers may be lost.

Periampullary cancer after surgery for esophageal cancer is just a typical disease. Since GDA gives off RGEA, which is preserved to maintain the circulation of gastric tube, and some small arterial branches directly to the pancreatic parenchyma, it is necessary to resect the GDA in radical operations on the periampullary cancer. Therefore, preserving the GDA may lead to disease recurrence after surgery for periampullary cancer.

Nagai et al. [2] performed a prospective study of 10 cases on GDA preservation in PPPD for periampullary cancer of the pancreas with confirmed absence of tissue invasion and lymph node metastasis in the vicinity of the GDA in a retrospective study of 8 patients who underwent PD. The study did not show direct invasion, lymphatic spread, or lymph node metastasis around the GDA, so PPPD with GDA preservation for periampullary cancer with confined tumor was strongly advocated. In addition, they posited that the operation could prevent massive hemorrhage of the GDA stump if a pancreatic fistula occurred. However, their case report did not include any pancreatic cancer patient.

Following a PubMed search using the keyword “PD after esophageal cancer”, 11 case reports were found, 7 of which were by Japanese authors (Table 1). In 5 of the cases [[3], [4], [5], [6], [7]], the primary disease was pancreatic head cancer and bile duct cancer in 2 cases each and metastatic renal cell carcinoma in 1 case. The prognosis was clearly described for 3 cases with 1 patient each dying in less than a year, surviving over a year and surviving over 14 months.

**Table 1:** Published case reports of pancreatoduodenectomy after esophagectomy for esophageal cancer.

Authors	Published year	Age	Gender	Disease	Timing of two operations	Years after esophageal op.	Preserved vessels	Prognosis	Appendix	Refs. No.
Our case		79	M	PHC	Metachoronous	11	RGEA, RGEV, RGA, RGV	5y2m, recurrent free, alive		
Okimoto et al.	2014	70	M	BDC	Metachoronous	10	RGEA, RGEV, RGA, RGV	Not described		[3]
Nandy et al.	2014	70	M	PHC	Metachoronous	3	Not described	Dead, <1y		[4]
Addeo et al.	2011	73	M	mRCC	Metachoronous	6	RGEA, RGEV	Not described		[5]
Fraguilidis et al.	2011	50	M	PHC	Metachoronous	13	RGEA	1y2m, liver metastasis, alive		[6]
Ikeda et al.	2006	61, 63	M	BDC	Metachoronous	10	RGEA, RGEV, RGV	1y, recurrent free, alive		[7]
Inoue et al.	2014	72	M	PHC	Metachoronous	10	RGEA, RGEV	6 m, recurrent free, alive	Reconstruction of RGEA to GDA, RGEV to L. renal vein.	[8]
Okochi et al.	2015	70	M	PHC	Metachoronous	5	RGEA	8m, alive	RGEA, resected and reconstructed to R. branch of MCA.	[9]
Nagano et al.	2005	55	M	PVC	Metachoronous	17	RCA, MCA, MCV	Not described	Post-DG, esophagectomy with TG. Colonic interposition.	[10]
Gyorki et al.	2011	58	M	pNET	Metachoronous	0.5	LGA, LGV, MCA, MCV	Not described	PHC and EC, found simultaneously. GDA and RGEA, resected.	[11]
Uehara et al.	2004	57	M	IPMN	Metachoronous	2	RGEA, RGEV, RGA	Not described		[12]
Belyaev et al.	2009	54	F	CP	Synchronous	0	None	2y6m, alive	TG, colonic interposition.	[13]
Kurosaki et al.	2000	72	M	IPMN	Synchronous	0	RGEA, RGEV	5y, recurrent free, alive		[14]

PHC; pancreas head cancer, BDC; bile duct cancer, mRCC; metastatic renal cell carcinoma, PVC; papilla vater carcinoma, pNET; pancreatic neuroendocrine tumor, IPMN; intraductal papillomucinous neoplasm, CP; chronic pancreatitis.

RGEA; right gastroepiploic artery, RGEV; right gastroepiploic vein, RGA; right gastric artery, RGV; right gastric vein, RCA; right colic artery, MCA; middle colic artery, MCV; middle colic vein, LGA; left gastric artery, LGV; left gastric vein, DG; distal gastrectomy, TG; total gastrectomy.

With the relatively higher risk of postoperative local recurrence of pancreatic head cancer compared to other periampullary cancers, more careful consideration is required for GDA preservation. Inoue et al. [8] reported that, for the pancreatic head cancer patient with suspected GDA invasion after surgery for esophageal cancer, the GDA was resected and microscopic vascular reconstruction was performed with anastomosis of the RGEA to the GDA stump and the RGEV to the left renal vein to maintain the blood circulation of the gastric tube.

Okochi et al. [9] also reported that they successfully anastomosed the cut end of the RGEA to the right branch of the middle colic artery (MCA) microscopically after RGEA resection because the pancreatic head cancer was adjacent to the vessel.

For colonic reconstruction after esophagectomy, different vessels are preserved. In the report by Nagano et al. [10], the right colic artery, MCA, and middle colic vein (MCV) were preserved in the cancer of papilla Vater patient, after esophagogastrectomy with colonic reconstruction following distal gastrectomy. Gyorki et al. [11] also reported the case of PD for pancreatic neuroendocrine tumor (pNET) after colonic reconstruction following esophagectomy. Although this case was diagnosed simultaneously as esophageal cancer and pNET, the surgery for each condition was performed at a different time. In the first surgery, which was for the esophageal cancer, the left gastric artery and vein were preserved, and the colon was used for reconstruction. In the second surgery, which took place 6 months after the esophageal surgery, PPPD was performed with resection of the GDA and preservation of the MCA and MCV using a well-planned strategy.

Uehara et al. [12], Belyaev et al. [13], and Kurosaki et al. [14] reported cases of periampullary benign diseases, such as intraductal papillary mucinous tumors and chronic pancreatitis. They succeeded in preserving the RGEA and RGEV (RGEV preservation was unclear in Ref. [8]) and recommended the procedure for the appropriate indications.

Even when the preceding operation was not esophagectomy with gastric tube reconstruction but proximal gastrectomy for cardiac cancer, Ikeda et al. [7] successfully performed PD with preservation of RGEA and RGEV to maintain blood circulation of the residual stomach.

Although these reported cases demonstrated the technical feasibility of PPPD with GDA-RGEA and RGEV preservation even with tumor invasion to GDA, PPPD can be performed with resection and reconstruction of GDA, the prognosis of which is still unclear especially in pancreatic head cancer patients.

Some reports found in PubMed did not present the prognosis of their cases. As shown in the table, the most favorable prognosis was 14 months for pancreatic head cancer. Patients of the other cases were all alive, but the survival times were shorter at 12, 8, and 6 months. In our case, the patient, who underwent PPPD with preservation of RGEA, RGEV, RGA, and RGV to maintain the blood circulation of gastric tube, survived with no disease recurrence for over 5 years.

## 4 Conclusion

PPPD after esophagectomy with gastric tube reconstruction is an effective surgical procedure whose indication may further increase when combined with microscopic surgery. However, as the survival rates in cases of GDA resection and preservation have not yet been confirmed to be the same, it is difficult to determine the validity of the procedure.

## Conflicts of interest

None.

## Sources of funding

None.

## Ethical approval

The approval has been given.

The ethics committee: Medical ethical committee of Showa Inan General Hospital.

The reference number No. 2018-09.

## Consent

Written informed consent was obtained from the patient for publication of this case report and accompanying images. A copy of the written consent is available for review by the Editor-in-Chief of this journal on request.

## Author contribution

Substantial contribution to the conception or design of the work; or the acquisition, analysis, or interpretation of the data for the work: TO.

Drafting the manuscript or critically revising it for important intellectual consent: TO.

Final approval of the version to be published: TO, MY, HK, YK.

Agreement to be accountable for all aspects of the work in ensuring that questions related to the accuracy or integrity of any part of the work are appropriately investigated and resolved: MY, HK, YK.

All authors read and approved the final manuscript.

## Registration of research studies

This study is report of a case, so has not been registered.

## Guarantor

Takashi Orii, corresponding author of this article.

## Provenance and peer review

Not commissioned, externally peer-reviewed.
